# The Quack Graduate

**Published:** 1896-05

**Authors:** 


### Editorial Department.

F. E. DANIEL, M. D., Editor.
S. E. HUDSON, M. D., Managing Editor.
A. J. SMITH. M. D., Galveston, Associate Editor.

EDITORIAL STAFF:

PROF. J. E. THOMPSON, M. D., Texas Medical College, Galveston; Surgery.
PROF. WM. KEILLER, M. D., Texas Medical College, Galveston; Obstetrics and Gynecology.
PROF. DAVID CERNA, M. D., Texas Medical College, Galveston; Therapeutics.
PROF. A. J. SMITH, M. D., Texas Medical College, Galveston; Medicine
DR. R. H. L. BIBB, City of Mexico; Foreign Correspondent.

Official organ of the West Texas Medical Association, the Houston District Medical Association, the Austin District Medical Society, the Galveston County Medical Society, and several others

The Quack Graduate.—That there are quacks in the profession as well as out of it, is well known. The possession of a diploma is no evidence, in every case, that its owner is not a quack. There are quacks in the State Medical Association. The only difference between them and the other fellow is, generally, they are not found out. When a college issues a diploma, the faculty have no guarantee that the one who receives it will not resort to unprofessional methods; and it seems to me that the Hippocratic oath should be administered to every graduate. It is generally understood that a college faculty have the right to revoke a diploma for unprofessional conduct; but too often the unprofessional conduct does not come to their knowledge; and should a physician report it against the one offending, his motives are impugned, and he is accused of jealousy; hence, many physicians who know that one with a diploma, and a legal right to practice, is practicing quackery, will not interfere.

In this connection, we cite a flagrant case by way of illustration. A valued correspondent sends us a card, of which the following is a verbatim copy—barring only the name of the two colleges and the "doctor," and the locality. The two colleges are in good standing—none better. It may be that this party is a fraud altogether, and that he has no diploma from either of the colleges; but here is what he is "doing it on." The writer has called the attention of the faculty of both colleges mentioned to the claim of this party that he has their diplomas; I have sent each a copy of his card; and by the next issue of the Jour-

NAL we will be in position to say whether or not he has the diplomas as claimed; and if he has, having forfeited it by such flagrant acts, we will see if the schools issuing these licenses can not revoke them.

This party, if he has a diploma, is beyond the reach of prosecution; he has complied with the law. Here is the card:

| GRADUATED AT | GRADAUTED AT |
|---|---|
| ——— UNIVERSITY, | ——— UNIVERSITY, |
| 1886 and '87. | 1892 and '93. |

Three Years Hospital Experience.
Four Years City and County Practice.
Have Taken a Special Course of Study in Chronic Diseases of Women.

I am 35 years of age, and feel that I am qualified to practice medicine in all of its branches intelligently, and having decided to make —— —— my future home, I shall stand on my own merits and practice medicine, at the following prices:

$1.00 for the first mile, and 50 cts. for each additional mile. At night I will charge $2.00 for first mile, and 50 cts. for each additional mile—Furnish my own medicine. Call visits $1.00. Obstetrical cases, $7.00 each; and all other work in proportion. I mean business. I have come to stay.

Respectfully,
—. —. ———, M. D.

—— ——, Texas.

. . . . . . .

In this connection, our attention has been called to the fact that in the lower part of the State, in a prosperous section, where there is an excellent medical contingent, there are practicing medicine three parties—one a negro—on so-called "diplomas," issued by the "Texas Health College," alleged to be located at Mound City, Coryell county. This is the work of the notorious "Orin Robinson." Our correspondents send us copies of these documents—which have been duly recorded—and ask what can be done. The attention of the attorney-general has been called to the matter, and some steps will be at once taken to prosecute the holders of such documents. There is no college, nor semblance of a college, at Mound City, and never has been, and the pretense is a fraud, notwithstanding a charter was issued to Orin Robinson, and his associates, in about 1885, for such college. They issued diplomas, but never taught;—sold them to whoever would buy.

They can be gotten at, and steps are being taken to have them prosecuted.

. . . . . . .

We have another case of bogus medical college to write up in next issue.

